# The Application of Manganese Complexes with Some Tetraazamacrocycles Immobilized in a Nafion Layer on a Glassy Carbon Electrode in Anodic Heterogenic Electrocatalysis

**DOI:** 10.3390/molecules31050800

**Published:** 2026-02-27

**Authors:** Danuta Tomczyk, Piotr Seliger

**Affiliations:** 1 University of Lodz, Department of Inorganic and Analytical Chemistry, Tamka 12, 91-403 Lodz, Poland; 2 University of Lodz, Department of Chemistry Teaching, Tamka 12, 91-403 Lodz, Poland; piotr.seliger@chemia.uni.lodz.pl

**Keywords:** Nafion, manganese complex, cyclam, modified electrode, electrocatalysis

## Abstract

Modified electrodes were obtained by immobilizing Mn^3+^ complexes with the following tetraazamacrocycles (1,4,7,10-tetraazacyclododecane ([12]aneN_4_), 1,4,8,11-tetrazacyclotetradecane ([14]aneN_4_), 1,4,7,11-tetrazacyclotetradecane (*iso*[14]aneN_4_), and 1,4,8,12-tetrazacyclopentadecane ([15]aneN_4_) in a Nafion film on the surface of a glassy carbon electrode (GCE). Based on spectroelectrochemical, chronopotentiometric, and chronoamperometric studies, oxidation of mononuclear complexes to dinuclear di-*μ*-oxo complexes of Mn^3+^ and Mn^4+^ was observed, and the mechanism and influence of Nafion on this process were determined. On the basis of voltammetric and chronocoulometric studies, the electroactivity, stability, and diffusion rates of such modified electrodes were demonstrated. Based on voltammetric and chronocoulometric studies, their electrocatalytic properties were analyzed in relation to the oxidation of model compounds used in this type of research, namely, ascorbic acid, glycolaldehyde, and glycolic acid.

## 1. Introduction

The presence of manganese ions in the biosphere has influenced the development of research into compounds of this element. Manganese ions are found in the active redox centers of many enzymes and in the OEC of PS II [1,2], but their role in these systems is still an active area of research. Hence, many synthetic compounds have been and continue to be studied in terms of the analysis of the function and structure of the active centers of natural systems. The OEC of PS II has been analyzed most intensively, and due to the proposed models of its active center, attempts are being made to synthesize and study multinuclear complexes at high oxidation states of Mn ions, containing, among others, O^2−^, O_2_^2−^, and CH_3_COO^−^ ions as bridging ligands [3,4,5]. Biocatalytic research is complemented by a wide range of studies on the use of manganese complexes in organic, regio-, chemo-, and stereoselective catalysis [6,7].

However, problems with separating the catalyst from the reaction products and regenerating its active form, as well as the fact that many of these complexes in homogeneous aqueous solutions do not exhibit catalytic activity due to the thermodynamic instability of the Mn^3+^, mean that both biochemical and chemical catalysis are shifting towards the study of heterogeneous systems [8,9,10,11]. Catalysts are immobilized in inorganic materials [12,13], redox polymers [14,15], and ion exchange polymers [16,17] to partially separate them from the aqueous environment, thus increasing the stability of the Mn^3+^. This field of research is completed by studies on the use of modified electrodes as sensors [18,19,20].

The aim of our research was to obtain stable complexes of manganese ions with a high oxidation state in a polymer film to assess the possibility of use in heterogeneous electrocatalysis of organic compound oxidation. Model compounds (ascorbic acid, glycolic acid, and glycolaldehyde) were selected as substrates for this type of research due to their well-known and well-documented oxidation mechanism, particularly in the case of ascorbic acid. The use of a polymer environment gave hope for an increase in the thermodynamic stability of the studied complexes by partially separating them from the aqueous medium.

Tetraazamacrocycles ([12]aneN4, [14]aneN4), *iso*[14]aneN4) and [15]aneN4 were selected as ligands due to their macrocyclic effect, which stabilizes thermodynamically unstable manganese ions at the +III oxidation state, as well as the fact that many of them form stable mono- and dinuclear complexes with Mn^3+^ and Mn^4+^ in both anhydrous and aqueous environments [21,22,23,24,25,26,27,28]. The selection of ligands with different coordination cavity sizes aimed to enable the assessment of the influence of this parameter on the stabilization of manganese complexes in the polymer film.

The polymer used to modify the glassy carbon electrode (GCE) was the ion-exchange polymer Nafion^®^117. It is a cation-exchange polymer that becomes electroactive as a result of the exchange of sulfonic acid groups for cationic complexes. The sulfonic acid groups in Nafion are highly acidic as a result of their connection to a strongly electron-accepting fluorocarbon chain. The choice of polymer was dictated by its stability in aqueous solutions, its resistance to oxidation, good adhesion to the electrode surface, the ability to bind large complex cations, and its use in catalytic studies [29,30,31,32,33]. In our previous papers, we reported on electrodes modified with this polymer with immobilized complexes derived from phenanthroline [34] and electrodes modified with conductive polymers derived from salen-type complexes [35,36].

## 2. Results and Discussion

### 2.1. Properties of Complexes

**Scheme 1:**
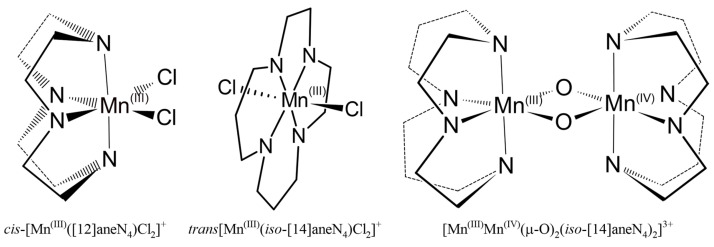
Structural formula of *cis*-[Mn^III^([12]aneN_4_)Cl_2_]+ and examples of formulas of the mononuclear trans-pseudo-octahedral complex of Mn^3+^, as well as dinuclear di-*μ*-oxo complexes of Mn^3+^ and Mn^4+^ with *iso*-[14]aneN_4_ macrocycle.

The synthesis of mononuclear Mn^2+^ complexes with the studied ligands was carried out by adding an ethanol solution of MnCl_2_ to ethanol solutions of ligands [28]. After acidification of the mixtures, pink solids were obtained, which after a short time changed into light green Mn^3+^ complexes with a *trans*-pseudo-octahedral structure (*trans*-[Mn^III^([14]aneN_4_)Cl_2_]Cl), *trans*-[Mn^III^(*iso*[14]aneN_4_)Cl_2_]Cl, and *trans*-[Mn^III^([15]aneN_4_)Cl_2_]Cl (Scheme 1). This type of isomerism is evidenced by characteristic features of electron spectra, i.e., a band at the border between near-UV and VIS [37] (e.g., for *trans*-[Mn^III^([14]aneN_4_)Cl_2_]Cl—342 nm, Figure S1, ESI, dotted line) and a weak band at 710 nm, corresponding to spin-allowed *d*-*d* transitions in high-spin Mn^3+^ complexes. Bands with higher absorption in the UV range (for *trans*-[Mn^III^([14]aneN_4_)Cl_2_]Cl 271 nm) indicate LMCT transitions.

In contrast, in the case of the *cis*-[Mn^III^([12]aneN_4_)Cl_2_]Cl complex, after acidification, the color remains pink due to *cis*-symmetry (Scheme 1). This is also evidenced by the electron spectrum [37], in which the electron bands are shifted towards shorter wavelengths (Figure S1, ESI, solid line, 304 nm). The reason for the different symmetry of the *cis*-[Mn^III^([12]aneN_4_)Cl_2_]Cl complex may be the smallest coordination cavity size of this ligand, which makes it difficult for Mn^3+^ to enter this space, as indicated by the same symmetry of the complex with smaller Co^3+^ [38].

The *cis-* or *trans*-symmetry of octahedral complexes is also evidenced by IR spectra in the range of deformation vibrations of amino groups (930–840 cm^−1^) and methylene groups (830–790 cm^−1^) [39]. *Trans*-octahedral complexes, regardless of the type of ligand and central ion, are characterized by one or two bands in the range of deformation vibrations of amino groups (e.g., for *trans*-[Mn^III^([14]aneN_4_)Cl_2_]Cl, 880 cm^−1^, Figure S2, ESI) and one in the range of methylene group vibrations (e.g., for *trans*-[Mn^III^([14]aneN_4_)Cl_2_]Cl, 807 cm^−1^, Figure S2, ESI). In contrast, *cis-*symmetric complexes are characterized by four or five bands in the range of amine group deformation vibrations (for *cis*-[Mn^III^([12]aneN_4_)Cl_2_]Cl: 912, 877 and 859 cm^−1^) and two in the range of methylene group vibrations (for *cis*-[Mn^III^([12]aneN_4_)Cl_2_]Cl: 823 and 791 cm^−1^, Figure S2, ESI).

The solubility of the complexes in water is very good, with the exception of *trans*-[Mn^III^([15]aneN_4_)Cl_2_]Cl, which dissolves after a longer period of time, after several dozen minutes. Aqueous solutions of mononuclear complexes of Mn^3+^ in a neutral medium show a strong tendency to form dinuclear di-*μ*-oxo complexes of Mn^3+^ and Mn^4+^, in which the manganese ions [Mn^III^(*μ*-O)_2_Mn^IV^]^3+^ hold the ligands in *cis*-pseudo-octahedral coordination [21,28] (Scheme 1). In the group of mononuclear complexes, the least stable is the aqueous solution of the *cis*-complex *cis*-[Mn^III^([12]aneN_4_)Cl_2_]Cl, due to the type of symmetry facilitating the formation of dimers (already after ~2 weeks). The very high tendency of mononuclear complexes of Mn^3+^ with low oxidation potential (~0 V vs. SCE (all potentials in this manuscript refer to SCE) [28]) to form dimers with higher oxidation potential is advantageous due to the use of monomers in anodic electrocatalysis for a wider group of substrates.

### 2.2. Electroactivity of Modified Electrodes

**Figure 1:**
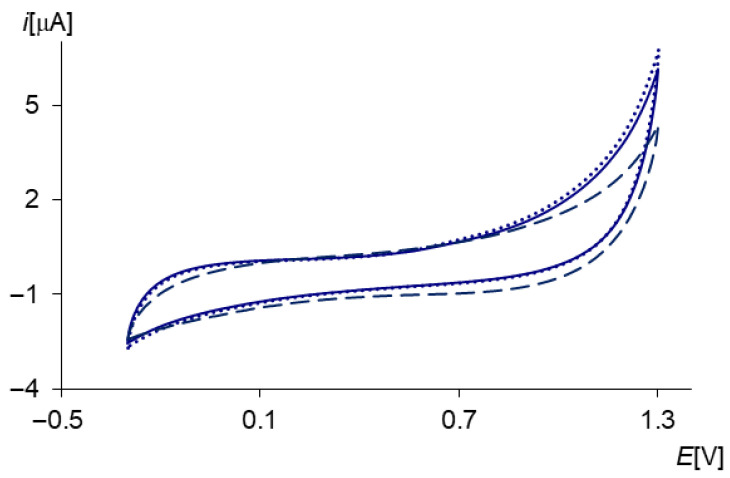
Cyclic voltammetry curves recorded on a glassy carbon disc electrode in KCl solution (*c* = 0.1 mol·dm^−3^): dotted line—uncoated electrode, dashed line—Nafion-coated electrode, solid line—Nafion-coated electrode with immobilized ligand ([14]aneN_4_) (*c* ~0.19 mol·dm^−3^), *v* = 0.05 V·s^−1^ vs. SCE.

**Figure 2:**
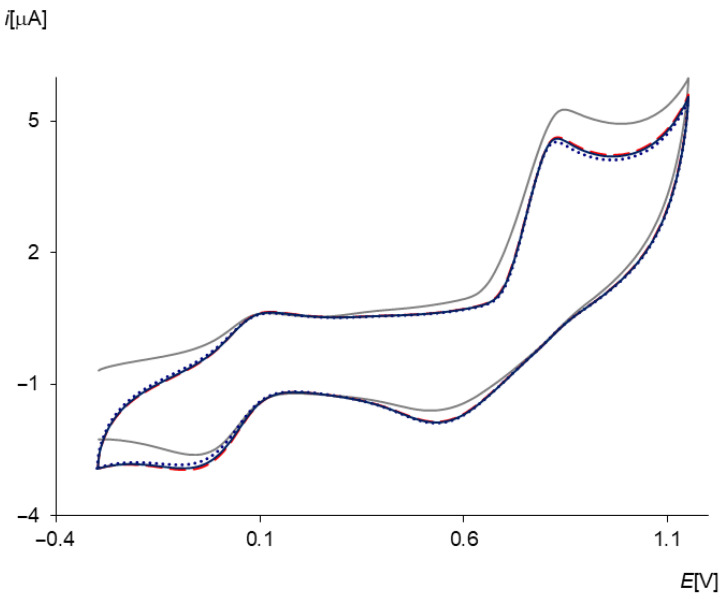
Cyclic voltammetry curves recorded on a glassy carbon disc electrode modified with the *trans*-[Mn^III^([14]aneN_4_)Cl_2_]Cl complex immobilized in Nafion (*c* = 0.062 mol·dm^−3^), gray line—1st cycle, dashed red line—2nd cycle, solid navy line—10th cycle and dotted navy line—30th cycle, *μ* = 0.1 (KCl), *v* = 0.05 V·s^−1^ vs. SCE.

Electrodes modified with Nafion and Nafion with immobilized ligands do not exhibit electroactivity in the investigated potential range (Figure 1). No peaks were observed on the voltammetric curves. The shape of the peaks on the voltammetric curves of electrodes modified with complexes (e.g., Figure 2), which differs from surface-type peaks, indicates diffusion from an unlimited space and thus thick films [40,41] and may reflect the high mobility of complexes. Such curves were observed for modified electrodes containing complexes in the Nafion film at all tested concentrations.

The electroactivity of the modified electrodes was studied using cyclic voltammetry in the potential range −0.3 to 1.25 V vs. SCE at 0.005 ≤ *v* ≤ 0.5 V·s^−1^.

Multiple recordings of cycles on an electrode modified with a complex immobilized in a Nafion film showed a very slight decrease in peak currents in subsequent cycles, starting from the second cycle, which indicated the stable nature of the electrodes obtained (Figure 2). The very rapid stabilization of the current was related to the preceding conditioning process of the modified electrode, which consisted of immersing it in a deoxygenated supporting electrolyte solution for 30 min. This resulted in the stabilization of the conductivity of the modified electrode, associated with the processes occurring in the contact between the film and the electrolyte solution. One of these processes was the movement of protons leaving the polymer environment as a result of the exchange of sulfonic groups for cationic complexes. Nafion is a highly sulfonated polymer in which there is one mole of sulfonic groups per ~1100 g of polymer; hence, it easily forms hydrophilic channels for proton transport and is characterized by high ion exchange capacity (IEC ~0.9–1.0 meq/g) [42]. In particular, it exhibits a very strong ability to bind large cations [32,40] due to the large diameter of the clusters (~4–5 nm) [43]. Comparable peak current values during the recording of successive cycles (Figure 2) indicate strong binding of the complexes we studied by this polymer.

The next process during conditioning of the modified electrode is the diffusion of anions into the electrolyte solution, in connection with maintaining the electro-neutrality of the film. Another process occurring during electrode conditioning was the diffusion of supporting electrolyte cations into the polymer film, to an extent dependent on the saturation of the film by the complex and the associated exchange of H^+^ for K^+^. The key process facilitating the above is the diffusion of water molecules into the polymer structure. The slight differences between the second and subsequent voltammetric cycles indicate a sufficient level of film hydration and stabilization of electrode conductivity, which means that the electrode conditioning time was sufficient for the film to reach equilibrium.

The stability of the modified electrodes obtained is also demonstrated by the fact that there is no significant diffusion of complex ions from the film. The theory of electrode processes shows that the oxidation of a polymer film containing cationic manganese complexes and free K^+^, in connection with the maintenance of the condition of electro-neutrality, is accompanied by the diffusion of electrolyte anions into the film [44] or the diffusion of cations into the solution [45]. The second way of maintaining the condition of electro-neutrality mainly concerns the cations that diffuse the fastest and interact the least with sulfonic groups, i.e., K^+^ [45], but it does not eliminate the possibility of diffusion of complex cations from the film. However, taking into account the very slight differences between successive cycles, starting from the second cycle (Figure 2), it can be assumed that the diffusion of complex ions from the polymer does not occur to a significant extent. Otherwise, the differences would be greater. On the other hand, the reduction process, in connection with the maintenance of the condition of electro-neutrality, is accompanied by the diffusion of anions into the electrolyte solution or K^+^ cations into the film.

### 2.3. Analysis of Electrode Process Mechanisms

The electrode processes occurring on modified electrodes were studied using cyclic voltammetry, chronocoulometry, chronoamperometry, chronopotentiometry, and spectroelectrochemistry.

**Figure 3:**
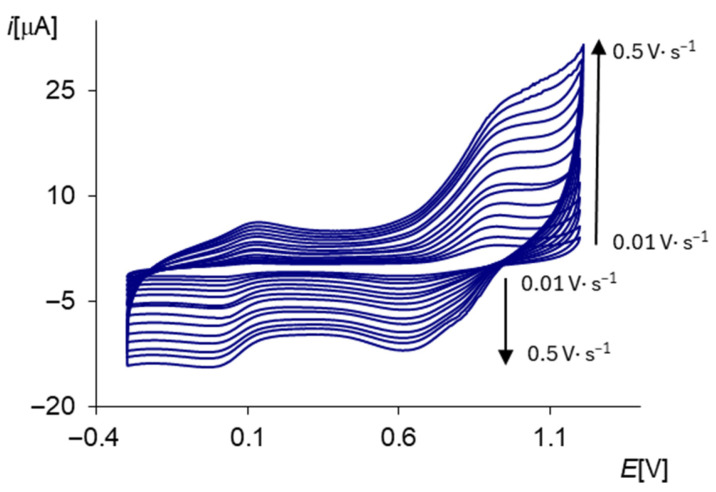
Cyclic voltammetry curves recorded on a glassy carbon disc electrode modified with the *trans*-[Mn^III^(*iso*[14]aneN_4_)Cl_2_] complex immobilized in Nafion (*c* = 0.062 mol·dm^−3^), *μ* = 0.1 (KCl), at *v*: 0.01, 0.03, 0.05, 0.07, 0.09, 0.12, 0.15, 0.20, 0.25, 0.30, 0.35, 0.40, 0.45, and 0.50 V·s^−1^, vs. SCE.

**Figure 4:**
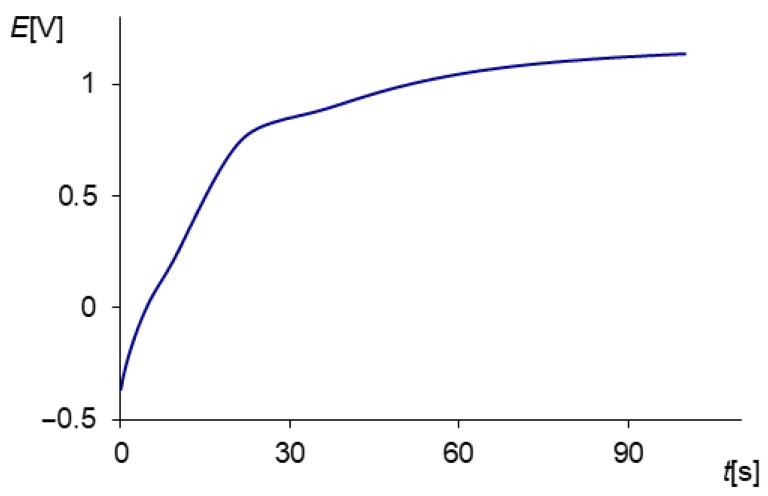
Chronopotentiometric curve recorded on a glassy carbon disc electrode modified with the *trans*-[Mn^III^(*iso*[14]aneN_4_)Cl_2_]Cl immobilized in Nafion, (*c* = 0.062 mol·dm^−3^), *μ* = 0.1 (KCl), *i* = 0.4 μA vs. SCE.

**Figure 5:**
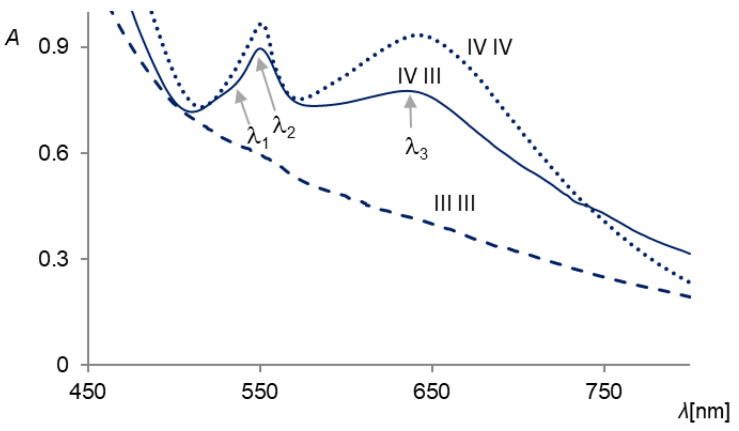
UV–VIS spectra recorded on a glassy carbon disc electrode modified with the *trans*-[Mn^III^(*iso*[14]aneN_4_)Cl_2_] complex immobilized in Nafion (*c* = 0.062 mol·dm^−3^): solid line—before electrolysis with controlled working electrode potential; dashed line—after electrolysis at 0 V vs. SCE; and dotted line—after electrolysis at 1 V.

Electrodes modified with Mn^3+^ complexes with the investigated ligands (*trans*-[Mn^III^([14]aneN_4_)Cl_2_]Cl, *trans*-[Mn^III^(*iso*[14]aneN_4_)Cl_2_]Cl, *cis*-[Mn^III^([12]aneN_4_)Cl_2_]Cl, and *trans*-[Mn^III^([15]aneN_4_)Cl_2_]Cl) oxidize and reduce in a two-step process (Figure 3), analogous to behavior in aqueous solutions of mononuclear Mn^3+^ complexes in a neutral medium, after prolonged scanning [28]. The chronopotentiometric curves are poorly shaped but may also indicate two-step electrode processes (Figure 4). Based on previously published data [28] and spectroelectrochemical studies of modified electrodes done after electrolysis process with controlled working electrode potential (Figure 5), it was found that this process, in its first step, corresponds to the oxidation of dinuclear complexes of Mn^3+^ di-*μ*-oxo, which hold ligands in *cis-*pseudo-octahedral coordination to di-*μ*-oxo dimers of Mn^3+^ and Mn^4+^ (Figure 5, solid line) and to di-*μ*-oxo dimers of Mn^4+^ in the second oxidation step (Figure 5, dotted line).

The presence of di-*μ*-oxo Mn^3+^ and Mn^4+^ dimers in Nafion films is evidenced by the electron spectra of neutral films that have not undergone the electrode process (Figure 5, solid line). The broad low-energy band in the VIS-NIR range corresponds to transitions between manganese atoms at different oxidation states [46]. The band at wavelength *λ*_3_ indicates O → Mn^4+^ transitions [47], and the band at *λ*_2_ indicates *d-d* transitions in Mn^4+^ [48]. The inflexion at *λ*_1_ corresponds to *d-d* transitions in Mn^3+^ in binuclear complexes with mixed valence [49].

However, the oxidation of films containing dinuclear complexes of Mn^3+^ and Mn^4+^ di-*μ*-oxo to dimers of Mn^4+^ di-*μ*-oxo is evidenced by the electron spectra obtained after oxidation of the films at the oxidation potentials of the second step of the electrode process (Figure 5, dotted line). The spectra show a clear decrease in the characteristic absorption of dinuclear complexes with mixed valence in the VIS-NIR range at ~800 nm. There is also no inflexion at ~540 nm, corresponding to *d*-*d* transitions in Mn^3+^ in dimers with mixed valence of central ions. On the other hand, the bands associated with *d*-*d* transitions in Mn^4+^ (*λ*_2_) and LMCT O → Mn^4+^ transitions (*λ*_3_) have increased, indicating a higher concentration of Mn^4+^ in the dimer [21].

The reduction in Nafion films containing dinuclear complexes of Mn^3+^ and Mn^4+^ to dimers of Mn^3+^ is evidenced by the absence of bands in the electron spectra obtained after the reduction in films at the reduction potentials of the first step of the electrode process (Figure 5, dashed line) [21]. After this process, the films became colorless. The nature of the spectra and the color of the films allowed us to exclude the decomposition of dimers into Mn^3+^ monomers. (For mononuclear Mn^3+^ complexes, characteristic bands would be observed in the electron spectra, and the films would be green [25,28,50,51].) The oxidation of reduced films, i.e., dinuclear complexes of di-*μ*-oxo Mn^3+^ at the oxidation potential of the first step of the electrode process, resulted in the formation of green-colored films, and the electron spectra showed the presence of di-*μ*-oxo Mn^3+^ and Mn^4+^ dimers in the films.

The possibility of obtaining a dinuclear di-*μ*-oxo complex of Mn^3+^ and Mn^4+^ ions in the film, maintaining ligands in *cis-*pseudo-octahedral coordination, from a mononuclear complex with a *trans-*pseudo-octahedral structure is associated with the prior exchange of axial Cl^−^ ligands for water and isomerization and deprotonation. As shown in [28], this process occurs in aqueous solutions during the electrode process. The exchange of Cl^−^ ligands for H_2_O takes place under current-free conditions. In aqueous solutions of mononuclear complexes, the two-step process, indicating the presence of dinuclear complexes, is observed only after recording several voltammetric cycles [28]. However, in the case of voltammograms recorded for complexes immobilized in Nafion, the presence of a two-step process was observed, indicating the presence of dinuclear complexes from the first cycle. This result indicates that the conditioning of the modified electrode in KCl solution not only promotes processes related to the movement of protons, electrolyte ions, and water molecules but also leads to the dimerization of mononuclear complexes. The recording of voltammetric curves without prior electrode conditioning led to the same mechanism of obtaining a dinuclear system that occurred during the recording of voltammetric curves in aqueous solutions of these complexes.

The possibility of dimerization in Nafion can be explained by the structure of this polymer. Nafion is a polymer characterized by the strong acidity of sulfonic groups caused by the inductive effect of the electron-accepting fluorocarbon chain. The morphology of the Nafion film is not clearly defined. Most likely, two models are the most probable. The two-phase model proposed by Gierke and Hsu [52], in which hydrophilic clusters containing sulfonic groups, their counterions, supporting electrolyte ions, and water are connected to each other by short, narrow channels capable of transporting charge and ions. The clusters are dispersed in the hydrophobic fluorocarbon phase of the polymer and are formed as a result of repulsion between the charged groups and the chain with a low dielectric constant. In the three-phase model proposed by Yeager and Steck [53], the hydrophilic clusters are located in a transition phase that contains a part of the polymer side chains, a small number of sulfonic groups, counterions, water, and a large area of empty space. The transition phase with clusters located within it is contained in the hydrophobic fluorocarbon phase. The structure of Nafion is not porous, and, therefore, the presence of a solvent is necessary to form clusters and channels that enable charge transport. Due to this structure of Nafion, the local concentration of complexes in clusters is several times higher than the average concentration in the entire polymer mass, which justifies the possibility of dimerization, which is favored by a higher concentration of monomers. In addition, the protons responsible for the hydrolysis of oxygen bridges leave the Nafion medium, and water molecules—the source of oxygen bridges—penetrate the polymer structure, further promoting dimerization. The data in the literature shows the dimerization of certain cations (Ti^3+^, Cu^2+^, Fe^3+^, VO^2+^) in Nafion films, while the same ions in aqueous solutions do not undergo such processes, indicating that the Nafion environment is a medium conducive to dimerization [52,54,55,56].

Based on the analysis of voltammetric curves, it can be concluded that the first step of the electrode process occurs according to a simple mechanism E. The currents of both peaks increase with increasing *v*. The ratio of anodic and cathodic peak currents at each scan rate change is less than one, but comparable. The differences in peak potentials at 0.005 ≤ *v* ≤ 0.01 V·s^−1^ reach values of 0.057 V, indicating single-electron processes. Above a scan rate of 0.02 V·s^−1^, the process is quasi-reversible, 0.057 < Δ*E* < 0.150 V.

**Figure 6:**
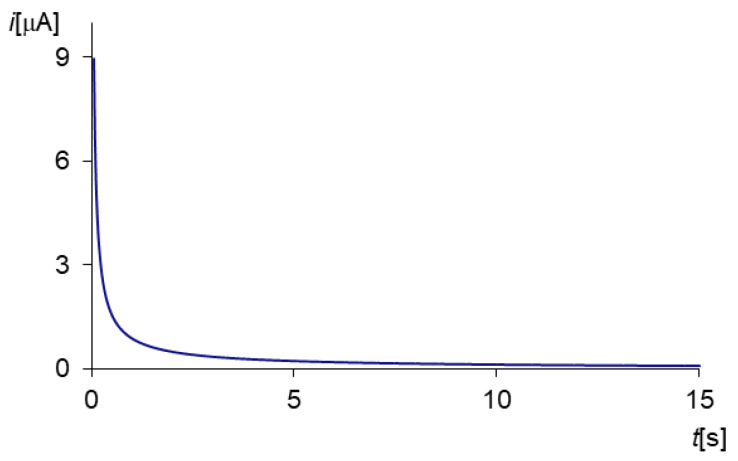
Chronoamperometric curve recorded for the second step of the oxidation process on a glassy carbon disc electrode modified with the *trans*-[Mn^III^(*iso*[14]aneN_4_)Cl_2_]Cl complex immobilized in Nafion (*c* = 0.062 mol·dm^−3^), *μ* = 0.1 (KCl), pulse time = 15 s, step potential: 0.5–1.0 V vs. SCE.

**Figure 7:**
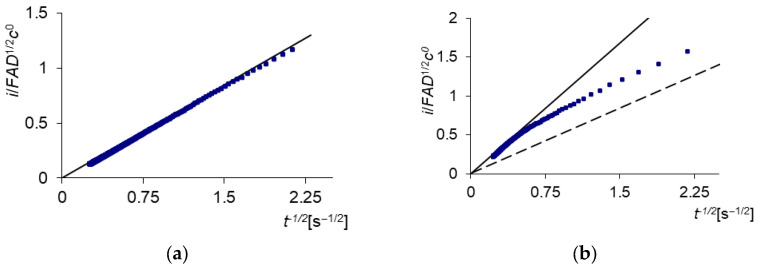
The *i*/*FAD*^1/2^*c*^0^ = f(*t*^−1/2^) dependence curves obtained from the chronoamperometric curves recorded on a glassy carbon disc electrode modified with the *trans*-[Mn^III^(*iso*[14]aneN_4_)Cl_2_] complex immobilized in Nafion (*c* = 0.062 mol·dm^−3^): (**a**) for the first step of the oxidation process, dotted navy line—experimental curve, solid black line—theoretical curve for *n* = 1, *μ* = 0.1 (KCl), pulse time= 15 s, step potential: −0.1–0.4 V vs. SCE; (**b**) for the second step of the oxidation process, shown in Figure 6, dashed black line—theoretical curve for *n*_1_ = 1, navy dotted line—experimental curve, solid black line—theoretical curve for *n*_1_ + *n*_2_ = 2.

The simple nature of the first step of the electrode process is confirmed by chronoamperometric studies [57] conducted for long pulse durations (Figure 6). The long pulse duration used allows for the analysis of mechanisms more complicated than the E mechanism. The measurement was performed using the double potential step method, from the initial value at which Faradaic currents did not flow to the final potential at which the concentration of the electroactive substance on the electrode surface reaches zero (from 0.1 V to ~0.4 V vs. SCE). The current recorded at this potential step corresponds to the oxidation of the dimer [Mn^III^Mn^III^(*μ*-O)_2_L_2_]^2+^ to [Mn^III^Mn^IV^(*μ*-O)_2_L_2_]^3+^, where L denotes the ligand. The slopes of the curves *i*/*FAD*^1/2^*c*^0^ = f(*t*^−1/2^) (where *F* is the Faraday constant, *A* is the surface area of the electrode, *D_ap_* is the apparent diffusion coefficient) (Figure 7a, dotted navy line), obtained on the basis of chronoamperometric curves and corresponding to one (Figure 7a, solid black line), indicate that the processes are related to the exchange of one electron and occur according to E mechanism and confirm the fact that dimerization takes place during the conditioning of the electrode in the electrolyte solution. If dimerization occurred only during the electrode process, the curves of the relationship *i*/*FAD*^1/2^*c*^0^ = f(*t*^−1/2^) would show a deviation from linearity in the range corresponding to long times, indicating an electrode process involving the exchange of less than one electron resulting from the CE mechanism.

The voltammetric curves of the second step of the electrode process are irreversible across the entire range of applied scan rates. The peak potential differences are greater than 0.220 V vs. SCE (Figure 3). The anodic peak currents are higher than the cathodic currents at a given potential rate. The currents of both peaks increase with increasing *v*. The increase in cathode peak currents is greater at higher scan rates. The potentials of the anode peaks shift towards higher potential values as the scan rate increases. The same direction of potential changes occurs for cathode peaks. In addition, at higher *v* on the reduction curves corresponding to the electron transfer process, there is an inflection. The transition coefficients decrease with increasing *v*. This nature of the voltammograms excludes a simple mechanism of the electrode process and may suggest a process that occurs according to the ECE mechanism. Then, in Figure 3, the presence of an inflection instead of a second reduction peak may indicate a fast chemical reaction, and the second step observed in the electrode process may reflect only the second electrode reaction of this mechanism.

The type of mechanism of the second step of the electrode process was determined based on chronoamperometric curves. The measurement was carried out in an analogous manner to that during the analysis of the first stage of the process, using the double potential step method, from 0.5 V to ~1.0 V vs. SCE. The difference was that the recording of the curves was preceded by electrolysis at the oxidation potential of [Mn^III^Mn^III^(*μ*-O)_2_L_2_]^2+^ dimer (~0.4 V vs. SCE) so that the current recorded during the analysis of the second step of the electrode process was related only to the oxidation of the [Mn^III^Mn^IV^(*μ*-O)_2_L_2_]^3+^ dimer. The electrolysis time was determined based on the recording of chronocoulometric curves for the first steps of electrode processes, with long pulse times, allowing the completely oxidized films to be obtained [58]. For example, for a complex concentration in films equal to 0.062 mol·dm^−3^, the pulse time was 80 s. No further increase in charge was observed at the end of the anodic sections of the chronocoulometric curves (Figure S3a, ESI). In this case, a clear negative deviation from linearity was marked on the *Q* = f(*t*^1/2^) curve (Figure S3b, ESI). For electrodes modified with a multilayer film, this characteristic of chronocoulometric curves is interpreted in the literature [40,59] as the effect of depletion of the electroactive material from the film modifying the electrode. This is the case when the thickness of the diffusion layer is limited by the thickness of the polymer, and the recorded process is typical for diffusion in a thin layer. In this situation, it can be assumed that after 80 s of electrolysis, the [Mn^III^Mn^III^(*μ*-O)_2_L_2_]^2+^ dimer was completely oxidized to the [Mn^III^Mn^IV^(*μ*-O)_2_L_2_]^3+^ dimer, and the concentration of the [Mn^III^Mn^IV^(*μ*-O)_2_L_2_]^3+^ dimer can be assumed to be equal to the initial concentration of [Mn^III^Mn^III^(*μ*-O)_2_L_2_]^2+^.

Chronoamperometric curves for the second steps of electrode processes, after electrolysis, were recorded for long pulse times at which diffusion remains linear, unrestricted by kinetics or migration, and is not limited by polymer thickness. The diagnostic criterion for this diffusion is the linear dependence of the function *Q* = f(*t*^1/2^), and Figure S3b, ESI, shows that it is ~0.5–15 s. The curves of the dependence of *i*/*FAD*^1/2^*c*^0^ = f(*t*^−1/2^) (Figure 7b, dotted navy line), whose slope depends on time, are typical of the ECE mechanisms [60]. For short pulse times, the nature of the process corresponds to the electron exchange associated with a single electrode process of the ECE mechanism. In Figure 7b, the experimental curve shows that the electron nature of the process is lower than one. It does not coincide with the theoretical curve (Figure 7b, dashed black line), calculated for a simple, single-electron electrode process, indicating that the process associated with the exchange of a single electron occurs only for very short times, shorter than the experimental conditions, and it also indicates a high value of the secondary reaction rate constant (*k_b_*). For longer times, the electron transfer rate of the reaction depends on the *k_b_* value. Only for long pulse times does the nature of the process correspond to the electron exchange associated with two electrode processes of the ECE mechanism, regardless of the *k_b_* value. In Figure 7b, for long times (~10 s, depending on the complex), the curve coincides with the theoretical curve calculated for a simple two-electron electrode process (Figure 7b, solid black line), which means that the product of the subsequent reaction also oxidizes with one electron. Some light on this ECE mechanism is shed by the research of Brewer et al. [21]. The authors showed that the [Mn^III^Mn^IV^(*μ*-O)_2_([14]aneN_4_)_2_]^3+^ in the first electrode reaction oxidizes to the [Mn^IV^Mn^IV^(*μ*-O)_2_([14]aneN_4_)_2_]^4+^ dimer, which then undergoes a chemical reaction with water, and the product of this reaction also undergoes single-electron oxidation [21].

### 2.4. Diffusion Coefficients and Surface Center Concentrations

The rate of charge transport through the polymer film, apart from the transition coefficients, is characterized by the diffusion coefficients of the electroactive substance.

Charge transfer through an ion-exchanging polymer film involves the coexistence of three processes: physical diffusion of the redox centers, electron transfer between them (self-exchange), and counterion movement to ensure electro-neutrality. Due to the high viscosity of the polymer, electron self-exchange is of significant importance in charge transfer, in contrast to that of a solution, where electron transfer is minimal. Although this is not typical diffusion, this entire complex process is called diffusion [61] and, as shown by Andrieux, Saveant [62], and Laviron [63], can be described by Fick’s laws. The measure of diffusion understood in this way is the apparent diffusion coefficient, *D_ap_*_._

The relative contributions of physical diffusion and electron self-exchange in charge transport were first described by Dahms [64] and Ruff [65] with the equation:

$$D_{ap}=D_{phys}+D_{e}=D_{phys}+\frac{k_{e}\delta_{e}^{2}c{}^{\circ}}{6}$$

where *D_phys_* is the physical diffusion coefficient, *D_e_* is the electron self-exchange coefficient, *k* is the electron self-exchange rate constant, and *δ_e_* is the distance between the centers of neighboring redox particles, which is the overall concentration of the redox centers in the film, and the factor 1/6 results from the three-dimensional nature of the process.

The diffusion coefficients of complexes immobilized in the Nafion film were determined on the basis of chronocoulometric studies. Measurements for the first step of the oxidation process were performed with pulse times recorded within the range of 8–20 s, with a potential jump from ~−0.01 V to ~0.04 V vs. SCE.

However, the diffusion coefficients for the second step of the electrode process, due to the appearance of this step according to the ECE mechanism, were determined on the basis of curves recorded for the shortest possible pulse times (0.004–0.5 s), allowing the linear relationship *Q* = f(*t*^1/2^) to be obtained, with a potential step from ~0.05 V to ~1.0 V vs. SCE. The pulse time used was determined based on Figure 7b, which shows that only with a very short pulse time (<<1 s) are the conditions for only the first electrode reaction of the ECE mechanism to occur.

The recording of chronoculometric curves to determine the diffusion coefficients for the second step of the electrode process was preceded by electrolysis in the diffusion layer at a potential of ~0.4 V vs. SCE. For example, for a complex concentration in the film of *c* = 0.062 mol·dm^−3^, electrolysis was carried out for a period of 80 s. For higher concentrations, it was carried out for longer, up to 400 s for a concentration of *c* = 0.316 mol·dm^−3^. The electrolysis time was determined each time on the basis of the recording of chronocoulometric curves for the first step of the electrode process, with long pulse times. The purpose of the electrolysis was to oxidize the [Mn^III^Mn^III^(*μ*-O)_2_L_2_]^2+^ dimer to [Mn^III^Mn^IV^(*μ*-O)_2_L_2_]^3+^ so that the charge during the recording of the curve for the second stage of the process corresponded only to the oxidation of the [Mn^III^Mn^IV^(*μ*-O)_2_L_2_]^3+^ dimer to [Mn^IV^Mn^IV^(*μ*-O)_2_L_2_]^4+^.

For all modified electrodes, the *D_ap_* values (Table 1) increase with increasing concentration of the complex in the film. This relationship indicates that charge transport occurs via the physical diffusion of ions and self-exchange of electrons. If charge transport occurred exclusively through physical diffusion of ions, the *D_ap_* values would be constant, regardless of the concentration of the complex in the film [66].

The nature of physical diffusion depends on the strength of the electrostatic interaction between redox centers and Nafion. Depending on the magnitude of this force, physical diffusion can be either free diffusion (10^−9^ < *D_phys_* < 10^−7^) or bound diffusion (10^−13^ < *D_phys_* < 10^−10^ cm^2^s^−1^) [67]. The diffusion coefficients of free metal ions are usually of the order of 10^−7^ cm^2^s^−1^. The order of the obtained *D_ap_* values (Table 1) indicates a type of diffusion between free diffusion and bound diffusion of complex ions [68].

The order of magnitude of the determined *D_ap_* values also confirms the hydrophilic nature of complex ions [66]. The *D_ap_* values of hydrophilic cations in the range of 10^−9^–10^−10^ are greater than those of hydrophobic cations (~10^−11^ cm^2^s^−1^) due to the cluster phase they occupy in the polymer and the charge transport that occurs by physical diffusion [66]. For large hydrophilic ions, physical diffusion may be difficult, and charge transport is also carried out by electron transfer between redox centers due to the fact that they can occupy both the cluster phase and the transition phase [66].

Thus, it can be concluded that despite showing the contribution of self-exchange to the total charge transport and the partial occurrence of associated diffusion, the obtained *D_ap_* values indicate a significant contribution of unassociated physical diffusion and may, therefore, suggest high mobility of the investigated complexes.

The diffusion coefficient values for all complexes with the same concentrations, both for the first step of the oxidation process and for the second, are similar. The dimers, [Mn^III^Mn^III^(*μ*-O)_2_([12]aneN_4_)_2_]^2+^ and [Mn^III^Mn^IV^(*μ*-O)_2_([12]aneN_4_)_2_]^3+^, show slightly higher values for these parameters. The reason for this is the smaller size of these ions, which facilitates physical diffusion in the polymer environment, which generally hinders this process [68]. The reason for easier physical diffusion may be the place that ions occupy in the Nafion structure. A review of the literature shows that small cations, such as Na^+^, occupy only the cluster phase due to strong electrostatic interactions with sulfonic groups [68], while large cations occupy both the cluster and transition phases [66]. For this reason, it can be assumed that the smallest of the studied dimers occupy the cluster phase to a greater extent, which is richer in water than the transition phase [52] and which, for this reason, facilitates physical diffusion, resulting in easier charge transport and higher *D_ap_* values.

On the other hand, the smallest Dap values characterize [Mn^III^Mn^III^(*μ*-O)_2_([15]aneN_4_)_2_]^2+^ and [Mn^III^Mn^IV^(*μ*-O)_2_([15]aneN_4_)_2_]^3+^, most likely due to the larger size of these ions. The larger size of the dimer may contribute to a smaller share of physical diffusion in the charge transport process because the larger the ion size, the more difficult this process is [68], which in turn may hinder charge transport. The less hydrophilic nature of *trans*-[Mn^III^([15]aneN_4_)Cl_2_]Cl compared to the others, as indicated by the more difficult solubility of this complex, may also contribute to the lowest Dap values of dimers formed by [15]aneN4. Hydrophilic complexes in Nafion mainly occupy the cluster phase but also the transition phase [66]. The less hydrophilic nature of the [Mn^III^Mn^III^(*μ*-O)_2_([15]aneN_4_)_2_]^2+^ and [Mn^III^Mn^IV^(*μ*-O)_2_([15]aneN_4_)_2_]^3+^ dimers compared to the others, as well as their larger size, may cause these ions to occupy the transition phase to a greater extent than the others, which contains less water than the cluster phase, hindering physical diffusion and resulting in the lowest *D_ap_* values for these dimers.

The indirect *D_ap_* values for ions derived from [14]aneN_4_ and *iso*[14]aneN_4_ may confirm the dependence of ion size on diffusion rate, resulting from the place occupied in the polymer structure.

The surface concentrations of active centers in the investigated polymer films were determined on the basis of voltammetric studies, only for the first step of the electrode process, due to the occurrence of the second step of this process according to the ECE mechanism. Based on the calculated charge that flowed during the first step of the process, using Faraday’s law, *Γ* = *Q*/*zFA*, the value of *Γ* was determined. Since none of the electrode polarization rates used produced surface peaks characteristic of thin films, on the basis of which the *Q* factor could be determined, and since the voltammetric peaks for the first step are poorly shaped, the *Γ* values obtained should be treated as estimates. For a given volume concentration, the surface concentrations for each complex are analogous and amount to *Γ* = 6.7 × 10^−10^ mol·cm^−2^ for a concentration of *c* = 0.062 mol·dm^−3^, 9.9 × 10^−10^ (*c* = 0.128), 1.5 × 10^−9^ (*c* = 0.193), 1.8 × 10^−9^ (*c* = 0.244), 2.2 × 10^−9^(*c* = 0.316), and 2.5 × 10^−9^ (*c* = 0.371).

### 2.5. Electrocatalytic Properties of Modified Electrodes

To determine the electrocatalytic properties of electrodes modified with the investigated complexes, voltammetric curves were recorded in the absence and presence of a substrates (ascorbic acid, glycolaldehyde, and glycolic acid) with a concentration of c = 10^−3^ mol·dm^−3^. In the investigated potential range, none of them reacted on the electrode modified with Nafion without the complex. The experiments were carried out for modified electrodes for all six surface center concentrations. In each case, there was a *Γ* << *c* substrate.

In the case of CE catalytic reactions, regardless of the type of chemical reaction C, the voltammetric curves recorded in the presence of a substrate at a given *v* show an increase in the anodic peak current and a decrease in the cathodic peak current, even to the point of its disappearance, compared to the curves obtained in the absence of a substrate. The nature of the voltammetric curves reflecting the above mechanism depends on the type of chemical reaction [69].

When the chemical reaction is irreversible, the curves are characterized by peaks similar to those of a reversible electrode process. As the constant rate of the chemical reaction increases, the peaks become less defined, and at high values of this parameter, only waves are obtained, whose current values do not depend on *v*.

However, when the chemical reaction is reversible, the shape of the voltammetric curves corresponds to the curves of the electrode process with a follow-up reaction, proceeding according to the EC mechanism. As the value of the constant rate of chemical reaction increases, the curves do not become wavy but retain peaks that become narrower and narrower. This process is called redox catalysis [69].

**Figure 8:**
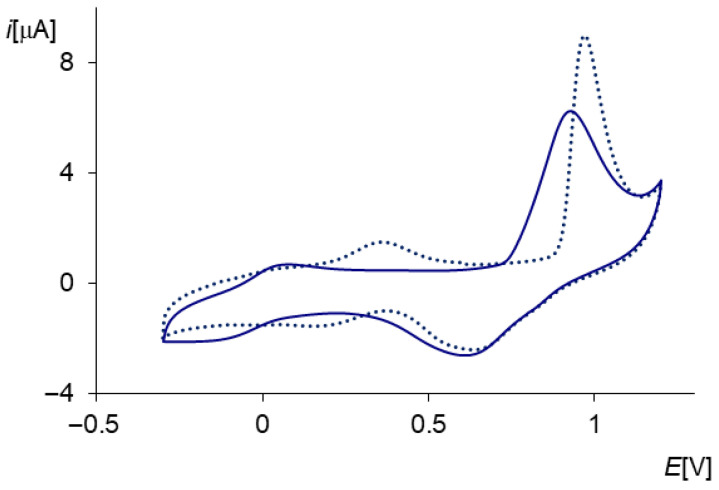
Cyclic voltammetry curves recorded on a glassy carbon disc electrode modified with the *trans*-[Mn^III^([15]aneN_4_)Cl_2_] complex immobilized in Nafion ((*Γ* = 6.7 × 10^−10^ mol·cm^−2^): solid navy line—in the absence of substrate, dotted line—in the presence of ascorbic acid (*c* = 10^−3^ mol·dm^−3^), *μ* = 0.1 (KCl), *v* = 0.05 V·s^−1^ vs. SCE.

For electrodes modified with all complexes, within the surface concentration range *Γ* = 6.7 × 10^−10^–2.5 × 10^−9^ mol·cm^−2^, the nature of the voltammetric curves recorded in the presence of a substrate, in relation to the curves recorded in the absence of a substrate, indicates catalytic activity achieved through redox catalysis (Figure 8 and Figure S4, ESI).

In the presence of a substrate, an increase in the values of anodic peak currents, a slight decrease in the values of cathodic peak currents, and a shift of anodic peak potentials towards higher values are observed (Figure 8, dotted line; Figure S4, ESI and red and blue lines; and Table 2). The processes catalyzed by the tested electrodes occur in each case at potentials more positive than the potentials of the mediators. The increase in peak potential values in the presence of a substrate may indicate a complicated catalytic process, resulting in the formation of a manganese complex with the substrate. The catalyzed reaction then occurs at a potential characteristic of this system.

**Figure 9:**
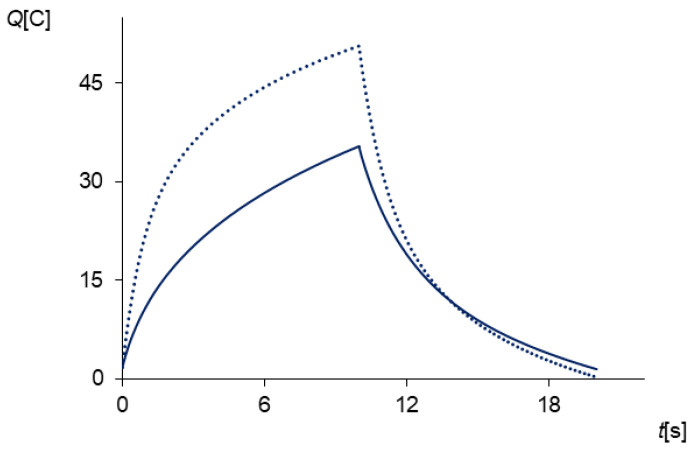
Chronocoulometric curves recorded on a glassy carbon disc electrode modified with the *trans*-[Mn^III^([15]aneN_4_)Cl_2_] complex immobilized in Nafion ((*Γ* = 6.7 × 10^−10^ mol·cm^−2^): solid line—in the absence of ascorbic acid, dotted line—in the presence of ascorbic acid (*c* = 10^−3^ mol·dm^−3^), *μ* = 0.1 (KCl), pulse time = 10 s, step potential: −0.1–1.2 V vs. SCE.

The catalytic effect is confirmed by chronocoulometric curves recorded in the presence and absence of the substrate. The increase in charge in the presence of the substrate (Figure 9, Table S1) is the result of the oxidation of a larger number of electroactive substances and confirms the process of electrocatalysis.

Another common feature of the modified electrodes is their lower catalytic activity towards glycolaldehyde and glycolic acid compared to their activity towards ascorbic acid (Table 2), as well as comparable electrocatalytic activity towards a given substrate, regardless of the concentration of the complex in the film (Table S2, ESI).

Comparing the values of anodic peak currents recorded in the presence of a substrate (*i_pa_*_S_) to those recorded in the absence of a substrate (*i_pa_*) (*i_pa_*_S_/*i_pa_*) (Table 2) shows that the best electrocatalytic properties are demonstrated by electrodes modified with the *cis*-[Mn^III^([12]aneN_4_)Cl_2_]Cl complex (the highest ipa S/ipa values), which may be related to the highest diffusion coefficients of [Mn^III^Mn^III^(*μ*-O)_2_([12]aneN_4_)_2_]^2+^ and [Mn^III^Mn^IV^(*μ*-O)_2_([12]aneN_4_)_2_]^3+^ dimers (Table 1). The *D_ap_* value, which characterizes the rate of charge transport, along with the rate of substrate penetration into the polymer film, also affects the quality of the electrocatalytic process. The best catalytic activity of this complex is also confirmed by the lowest values of the *i_pc_*_S_/*i_pc_* ratio (Table 2). Electrodes modified with *trans*-[Mn^III^([14]aneN_4_)Cl_2_]Cl and *trans*-[Mn^III^(*iso*[14]aneN_4_)Cl_2_]Cl complexes exhibit equally good electrocatalytic activity. Electrodes modified with the *trans*-[Mn^III^([15]aneN_4_)Cl_2_]Cl complex show slightly weaker activity, with the *i_pa_*_S_/*i_pa_* ratio showing the lowest value (Table 2). For each complex immobilized in the Nafion film, the same relationship between the values of *i_pa_*_S_/*i_pa_* and *D_ap_* can be observed. Higher values of *i_pa_*_S_/*i_pa_* correspond to higher values of *D_ap_*, indicating that faster kinetics of the electrode process favor a greater catalytic effect.

## 3. Materials and Methods

### 3.1. Ligands

Ligands (1,4,7,10-tetraazacyclododecane ([12]aneN_4_), 1,4,8,11-tetrazacyclotetradecane ([14]aneN_4_), 1,4,7,11-tetrazacyclotetradecane (*iso*[14]aneN_4_), and 1,4,8,12-tetrazacyclopentadecane ([15]aneN_4_) were from Merck-Sigma-Aldrich (Darmstadt, Germany).

Light green complexes (*trans*-[Mn^III^([14]aneN_4_)Cl_2_]Cl·2H_2_O, trans-[Mn^III^(*iso*[14]aneN_4_)Cl_2_]Cl·2H_2_O, *trans*-[Mn^III^([15]aneN_4_)Cl_2_]Cl·3H_2_O) and pink *cis*-[Mn^III^([12]aneN_4_)Cl_2_]Cl·3H_2_O were analyzed and obtained according to the synthesis procedure presented in [28].

*trans*-[Mn^III^([14]aneN_4_)Cl_2_]Cl·2H_2_O, Anal. Calcd. for C_10_H_24_N_4_Cl_3_Mn·2H_2_O: C, 30.20; H, 7.04; N, 14.09. Found C, 30.39; H, 7.00; N, 13.92.
*trans*-[Mn^III^(*iso*[14]aneN_4_)Cl_2_]Cl·2H_2_O, Anal. Calcd. for C_10_H_24_N_4_Cl_3_Mn·2H_2_O:C, 30.20; H, 7.10; N, 14.09. Found C, 30.42; H, 7.01; N, 13.91.
*trans*-[Mn^III^([15]aneN_4_)Cl_2_]Cl·3H_2_O, Anal. Calcd. for C_11_H_26_N_4_Cl_3_Mn·3H_2_O: C, 30.75; H, 7.45; N, 13.04. Found C, 31.18; H, 7.57; N, 13.08.
*cis*-[Mn^III^([12]aneN_4_)Cl_2_]Cl·3H_2_O, Anal. Calcd. For C_8_H_20_N_4_Cl_3_Mn·3H_2_O: C, 24.79; H, 6.71; N, 14.46. Found C, 23.65; H, 6.75; N, 14.51.

Nafion^®^117 (~5% in a mixture of lower aliphatic alcohols and water) and ethanol were purchased from Fluka (Darmstadt, Germany).

### 3.2. Modification of Electrodes

A glassy carbon disc electrode (MINERAL, Łomianki-Sadowa, Poland) with a surface area of *A* = 0.0175 ± 0.0002 cm^2^ was used for the modification. The exact surface area was determined based on voltammetric measurements recorded in a depolarizer solution with a known diffusion coefficient (K_3_[Fe(CN)_6_], *c* = 0.01 mol·dm^−3^ in KCl, *c* = 1 mol·dm^−3^) in the range from 0.3 to 0.8 V vs. SCE (Figure S5, ESI). The electrode surface area was calculated from the linear part of the slope of the *i_p_* = f(*v*^1/2^) relationship (Figure S6, ESI) described by the Randles–Ševčik equation for reversible processes.

In order to modify the electrode, a single-step modification method using complexes immobilized in a Nafion film was used [32]. The methodology for preparing the mixture consisted of combining an aqueous solution of a mononuclear manganese ion complex with an alcohol–water solution of Nafion in a volume ratio of 1:9. Complex solutions with the following concentrations were used for mixing with Nafion: 10^−2^, 2 × 10^−2^, 3 × 10^−2^, 4 × 10^−2^, 5 × 10^−2^, and 6 × 10^−2^ mol·dm^−3^. These complex concentrations and the ratio with Nafion proved to be the most advantageous in terms of the homogeneity and electroactivity of the film obtained on the electrode surface. The obtained mixtures retained the color of the initial complexes (light green except for pink for *cis*-[Mn^III^([12]aneN_4_)Cl_2_]Cl), regardless of their storage time. The mixtures were prepared 24 h before their use in measurements. The glassy carbon electrode was modified by applying 10 µL of the prepared mixture to its surface and leaving it for ½ hour for the solvents to evaporate. After this period, the electrode was conditioned by immersing it for 30 min in a deoxygenated KCl solution with a concentration of *c* = 0.1 mol·dm^−3^. After this process, the polymer films of all complexes were light green.

The modified electrodes obtained in this way remained stable for several days. Stored in air, after being prepared for measurements in the manner described, they gave comparable current responses when used 3–4 times for testing in the supporting electrolyte. Spectroscopic studies of solutions after conditioning polymer films in them did not reveal bands characteristic of complexes, thus indicating the absence of complexes from the films or possible diffusion of complexes from the films, but to an extent that did not affect the measurement results. In order to carry out these studies, 1 cm^3^ of Nafion mixtures of each complex were prepared. After drying the mixtures, 1 cm^3^ of KCl solution with a concentration of *c* = 0.1 mol·dm^−3^ was added to each mixture and left for 40 min, 10 min longer than the conditioning of the electrodes, to take into account the duration of the measurement procedures. After this period, the solutions from above the Nafion films were poured off and analyzed spectroscopically. In this way, solutions were obtained which, assuming only slight diffusion of complexes from the film (1%), would give concentrations that could be determined spectroscopically. The stability of the electrodes modified during and after electrochemical measurements was investigated spectroscopically by performing spectra of the films on the electrodes. Comparable bands indicated the stable nature of the electrodes, resulting from the good binding of the complexes with the sulfonic groups of Nafion. Due to the small amount of complex in Nafion films, spectroscopic studies of solutions after electrochemical measurements would not be reliable.

The concentrations of complexes in Nafion films were estimated based on the literature value of the density of Nafion film conditioned in a basic electrolyte solution [33] (*d* = 1.58 g·cm^−3^), assuming that conditioned films with complexes of the tested concentrations introduced into their structure do not significantly change the density. This assumption was made based on the comparable masses of conditioned Nafion films obtained by mixing Nafion with various complexes at different concentrations (differences ~4%, Table S3, ESI). In contrast, the masses of conditioned Nafion films containing the same amounts of different complexes showed even smaller weight differences, within ~1% (Table S3, ESI), which allowed us to assume that the concentrations of different complexes in conditioned films, formed from mixtures with the same amount of the introduced complex, are essentially equal and amount to, depending on the amount of the added complex, 0.062, 0.128, 0.193, 0.244, 0.316, and 0.371 mol·dm^−3^, respectively. Taking into account the low content of complexes (0.8–4.5%) in the total mass of conditioned films, it can be assumed that this assumption does not significantly affect the obtained concentration results. The measurements consisted of preparing 1 cm^3^ of Nafion mixtures of each complex (0.9 cm^3^ Nafion and 0.1 cm^3^ of a complex solution of a given concentration), leaving them to evaporate the solvent for 30 min, conditioning the resulting films for 30 min in a KCl solution (0.1 mol·dm^−3^), washing them with water after pouring off the KCl solution, and weighing them immediately after this process. The amount of mixture prepared (1 cm^3^), with a composition corresponding to the composition of the mixture applied to the electrode surface, allowed for accurate weighing of the film. Based on the masses of the conditioned films obtained, the volume of the conditioned films was calculated using the density value of such a film given in the literature [33]. Based on the known number of moles of complexes introduced into Nafion, the concentrations of complexes in the total mass of polymer films were calculated.

### 3.3. Instruments and Procedures

Spectroelectrochemistry measurements were performed using the spectroelectrochemical instrument SPELEC DROPSENS (DropSens, Asturias, Spain) with a UV–VIS reflection probe. The measurements were carried out in a cell containing reference and auxiliary electrodes immersed in a KCl solution (0.1 mol·dm^−3^), connected to a narrower part containing the working electrode and reflection probe in the same KCl solution. The distance between the probe and the electrode was 1 mm. The investigations of the modified electrodes were preceded by tests of an electrode covered with a Nafion film without polymer, in an electrolyte solution, as a background. The device automatically corrected the background.

FT-IR spectra were recorded using a Nicollet 8700 spectrometer (Lublin, Poland) and a standard KBr pellet technique (Lublin, Poland).

UV-Vis spectra of the complex solutions were performed on a Jasco V630 (Tokyo, Japan) spectrophotometer in a standard quartz (0.995 cm) cell.

Cyclic voltammetry, chronoamperometry, chronocoulometry, chronopotentiometry, and electrolysis with controlled potential were carried out using AUTOLAB PGSTAT 10 (Metrohm, Herisau, Switzerland) Eco Chemie in a three-electrode system.

Electrolysis with controlled potential was carried out at the values after the anodic and cathodic peak potentials.

Chronoamperometry and chronocoulometry were performed using a double potential step method from the initial value, at which no Faraday currents flow, to the final potential, at which the concentration of the electroactive substance on the electrode surface reaches zero, using different pulse times (10^−3^–400 s).

Chronopotentiometric measurements were performed using the single current step method with different step durations (40–100 s) and at different currents (±10^−7^–10^−5^A).

All electrochemical measurements were carried out in three electrodes system. A glassy carbon disk electrode (GCE) (MINERAL, Łomianki-Sadowa, Poland), unmodified or modified with polymer complexes in Nafion, was used as a working electrode. The SCE (MINERAL) connected to the bulk of the solution by a Luggin capillary was used as a reference electrode. A platinum plate was used as a counter electrode. Measurements were recorded in the presence of KCl aqueous solution (0.1 mol·dm^−3^) as the supporting electrolyte. The solutions were deoxidized with argon before measurements.

## 4. Conclusions

Spectroelectrochemical and voltammetric studies have shown that the Nafion medium promotes the dimerization of mononuclear Mn^3+^ complexes with the investigated tetraazamacrocycles, as well as the two-step oxidation of dimers [Mn^III^Mn^III^(*μ-*O)_2_(L)_2_]^2+^ to [Mn^III^Mn^IV^(*μ-*O)_2_(L)_2_]^3+^ and [Mn^IV^Mn^IV^(*μ-*O)_2_(L)_2_]^4+^ ions. The dimerization process occurs even under currentless conditions, and the peaks are better defined than in an aqueous solution and are observed from the first cycle.

Based on chronoamperometric studies, using the *i*/*FAD*^1/2^*c*^0^ = f(*t*^−1/2^) relationship, it was demonstrated that the first step of the anodic electrode process occurs according to the E mechanism, and the second according to the ECE one. The duration of the electrolysis preceding the second phase of the process was determined on the basis of chronocoulometric studies for long pulse times, allowing the depletion of the electroactive material from the film, modifying the electrode surface to be observed.

Chronocoulometric studies also allowed the kinetics of electrode processes to be determined by calculating diffusion coefficients. The relationship between the obtained *D_ap_* values and the concentrations of complexes in Nafion films indicates that charge transport occurs through physical diffusion of ions and electron self-exchange. However, the order of magnitude of the *D_ap_* values (5–9·10^−10^ cm^2·^s^−1^) corresponds to values characteristic of hydrophilic ions and indicates a type of diffusion between free and bound diffusion. The highest Dap values for *trans*-[Mn^III^([15]aneN_4_)Cl_2_], intermediate values for ions derived from [14]aneN_4_ and *iso*[14]aneN4, and the smallest for *cis*-[Mn^III^([12]aneN_4_)Cl_2_] indicate that the diffusion rate depends on the size of the ion, which may result from the place occupied by the ions in the polymer structure. The smallest ions occupy the most hydrated phase of the clusters to a greater extent, where diffusion occurs most easily, which results in higher *D_ap_* values. In contrast, the largest ions occupy the less hydrated transition phase to a greater extent, where diffusion is more difficult for this reason.

The electrocatalytic properties of the modified electrodes were determined based on voltammetric and chronocoulometric studies, conducted with respect to the oxidation of sample substrates (ascorbic acid, glycolaldehyde, and glycolic acid). Catalytic activity was observed through redox catalysis (peaks in the presence of a substrate are narrower than those recorded in the absence of a substrate) and a complex catalytic process (catalyzed processes occur at potentials more positive than the potentials for the mediators). Most likely, a manganese complex is formed with the substrate, which oxidizes at a potential other than that of the mediator.

Based on the obtained values of *i_pa_*_S_/*i_pa_*, *i_pc_*_S_/*i_pc_,* and *D_ap_*, it was found that faster kinetics of the electrode process promote a greater catalytic effect. The best electrocatalytic properties are observed for electrodes modified with the smallest complex, *cis*-[Mn^III^([12]aneN_4_)Cl_2_]Cl (the highest ipa S/ipa and lowest ipc S/ipc values, as well as the highest Dap values). The weakest electrocatalytic properties are shown by electrodes modified with the largest complex, *trans*-[Mn^III^([15]aneN_4_)Cl_2_]Cl (lowest *i_pa_*_S_/*i_pa_* values and lowest *D_ap_* values).

## Figures and Tables

**Table 1:** Apparent diffusion coefficients determined based on chronocoulometric studies for the oxidation process of Mn^3+^ complexes with the studied tetraazamacrocycles, immobilized in Nafion, on the surface of a glassy carbon disc electrode, *μ* = 0.1 (KCl) vs. SCE.

Complex	Concentration of the Complex in the Film *c* [mol·dm^−3^]	First Step Processes		Second Step Processes	
		*Q/t*^1/2^·10^6^	*D_ap_*·10^10^ [cm^2^s^−1^]	*Q/t*^1/2^·10^6^	*D_ap_*·10^10^ [cm^2^s^−1^]
*trans*-[Mn^III^([14]aneN_4_)Cl_2_]Cl	0.062	0.72	6.0	0.73	6.1
	0.128	1.53	6.7	1.53	6.7
	0.193 0.244 0.316 0.371	2.30 3.09 3.89 4.86	7.0 7.1 7.4 7.7	2.28 3.08 3.92 4.88	6.9 7.0 7.5 7.8
*trans*-[Mn^III^(*iso*[14]aneN_4_)Cl_2_]Cl	0.062	0.68	5.8	0.72	6.0
	0.128 0.193 0.244 0.316 0.371	1.58 2.31 3.12 3.87 4.79	6.6 6.9 7.2 7.3 7.5	1.56 2.28 3.12 3.88 4.81	6.5 6.8 7.2 7.4 7.6
*cis*-[Mn^III^([12]aneN_4_)Cl_2_]Cl	0.062	0.80	7.2	0.79	7.1
	0.128	1.66	7.4	1.66	7.4
	0.193 0.244 0.316 0.371	2.39 3.25 4.17 4.99	7.6 7.8 8.0 8.1	2.41 3.27 4.20 5.03	7.7 7.9 8.1 8.3
*trans*-[Mn^III^([15]aneN_4_)Cl_2_]Cl	0.062	0.69	5.7	0.71	5.8
	0.128 0.193 0.244 0.316 0.371	1.49 2.19 3.06 3.77 4.72	6.0 6.5 6.9 7.1 7.3	1.51 2.19 3.06 3.79 4.75	6.1 6.5 6.9 7.2 7.4

**Table 2:** Electrocatalytic activity of the complexes immobilized in Nafion (*Γ* = 6.7 × 10^−10^ mol·cm^−2^) on the surface of a conventional glassy carbon electrode towards selected substrates (*c* = 10^−3^ mol·dm^−3^), *μ* = 0.1 (KCl), *v* = 0.05 V·s^−1^ vs. SCE.

Complex	Substrate	First Step of The Process		Second Step of the Process	
		*i_pa_*_·S_/i_pa_ *	*i_pc_*_·*S*_/*i_p__c_* *	*i_pa_*_·S_/i_pa_ *	*i_pc_*_·*SR*_/*i_pc_* *
*trans*-[Mn^III^([14]aneN_4_)Cl_2_]Cl	ascorbic acid	1.63	0.87	1.68	0.85
	glycolic acid	1.59	0.88	1.61	0.87
	glycolic aldehyde	1.42	0.93	1.51	0.92
*trans*-[Mn^III^(*iso*[14]aneN_4_)Cl_2_]Cl	ascorbic acid	1.64	0.87	1.70	0.85
	glycolic acid glycolic aldehyde	1.56 1.48	0.87 0.91	1.62 1.53	0.86 0.89
*cis*-[Mn^III^([12]aneN_4_)Cl_2_]Cl	ascorbic acid	1.82	0.79	1.87	0.77
	glycolic acid	1.77	0.83	1.80	0.82
	glycolic aldehyde	1.72	0.85	1.74	0.84
*trans*-[Mn^III^([15]aneN_4_)Cl_2_]Cl	ascorbic acid	1.34	0.87	1.45	0.92
	glycolic acid glycolic aldehyde	1.28 1.26	0.89 0.91	1.40 1.38	0.93 0.94

* i_pa__·*S*_*/i_pa_*—values of anodic peak currents recorded in the presence of a substrate *(i_pa_*_·*S*_) to those recorded in the absence of a substrate (*i_pa_*); *i_pc_*·_*S*_*/i_pc_*—values of cathodic peak currents recorded in the presence of a substrate *(i_pc_*_·*S*_) to those recorded in the absence of a substrate (*i_pc_*).

## Data Availability

Data is contained within the article and Supplementary Material.

## Supplementary Materials

The following supporting materials can be downloaded at https://www.mdpi.com/article/10.3390/molecules31050800/s1. Figure S1: UV–VIS NIR spectra of 10^−3^ mol·dm^−3^: solid line—*trans*-[Mn^III^(iso[14]aneN_4_)Cl_2_]Cl, dotted line—*cis*-[Mn^III^([12]aneN_4_)Cl_2_]Cl in H2O, 0.093 cm cell. Figure S2: IR spectra of complexes *trans*-[Mn^III^([14]aneN_4_)Cl_2_]Cl and *cis*-[Mn^III^([12]aneN_4_)Cl_2_]Cl in the range of 790–930 cm^−1^, KBr pellet. Figure S3: (a) Chronocoulometric curve recorded for the first step of the electrode process on a glassy carbon disc electrode modified with *trans*-[Mn^III^(*iso* [14]aneN_4_)Cl_2_]Cl immobilized in Nafion (*c* = 0.062 mol·dm^−3^), *μ* = 0.1 (KCl), pulse time = 80 s, step potential: −0.1–0.4 V vs. SCE; (b) Q = f(t^1/2^) relationship curve for the oxidation process obtained from the chronocoulometric curve shown in Figure S3a. Figure S4: Cyclic voltammetry curves recorded on a glassy carbon disc electrode modified with the *trans*-[Mn^III^([15]aneN_4_)Cl_2_] complex immobilized in Nafion (*Γ* = 6.7 × 10^−10^ mol·cm^−2^): navy line—in the absence of substrate, red line—in the presence of glycolic acid, blue line—in the presence of glycolic aldehyde (substrate concentration, *c* = 10^−3^ mol·dm^−3^), *μ* = 0.1 (KCl), *v* = 0.05 V·s^−1^ vs. SCE. Figure S5: Cyclic voltammetry curves recorded on a glassy carbon disc electrode in solution, *c* = 0.01 mol·dm^−3^, *μ* = 1 (KCl) at *v*: 0.01, 0.02, 0.03, 0.04, 0.05, 0.06, 0.07, 0.08, 0.09, 0.10, 0.12, 0.15, 0.20, 0.25, 0.30, 0.35, 0.40, 0.45, and 0.50 V·s^−1^ vs. SCE. Figure S6: Dependence of cathode peak currents on the square root of the scan rate obtained from voltammograms recorded in K_3_[Fe(CN)_6_], *c* = 0.01 mol·dm^−3^, *μ* = 1 (KCl), glassy carbon disc electrode vs. SCE. Table S1: Electrocatalytic activity of the studied complexes immobilized in Nafion (*Γ* = 1.5 × 10^−9^ mol·cm^−2^) on the surface of a conventional glassy carbon electrode towards selected substrates (*c* = 10^−3^ mol·dm^−3^), *μ* = 0.1 (KCl), pulse time = 10 s, step potential: −0.1–1.2 V vs. SCE, determined on the basis of chronocoulometry. Table S2: Electrocatalytic activity of the *trans*-[Mn^III^(*iso*[14]aneN_4_)Cl_2_]Cl complex immobilized in a Nafion film on the surface of a conventional glassy carbon electrode against substrate, depending on the complex concentration in the polymer film, *μ* = 0.1 (KCl), *v* = 0.05 V·s^−1^ vs. SCE. Table S3: Masses of conditioned Nafion films with complexes immobilized in their structures.
