# Supplementary material for: The Application of Manganese Complexes with Some Tetraazamacrocycles Immobilized in a Nafion Layer on a Glassy Carbon Electrode in Anodic Heterogenic Electrocatalysis
Source: Molecules. 2026 Feb 27;31(5):800. doi: 10.3390/molecules31050800 (PMC12985962; doi:10.3390/molecules31050800)
Supplement: Supplementary file 1 [file molecules-31-00800-s001.zip › molecules-4146521-supplementary.pdf]

Supplementary materials

The Application of Manganese Complexes with some Tetraazamacrocycles Immobilized in Nafion Layer on Glassy Carbon Electrode in Anodic Heterogenic Electrocatalysis

D. Tomczyk, P. Seliger

Figures

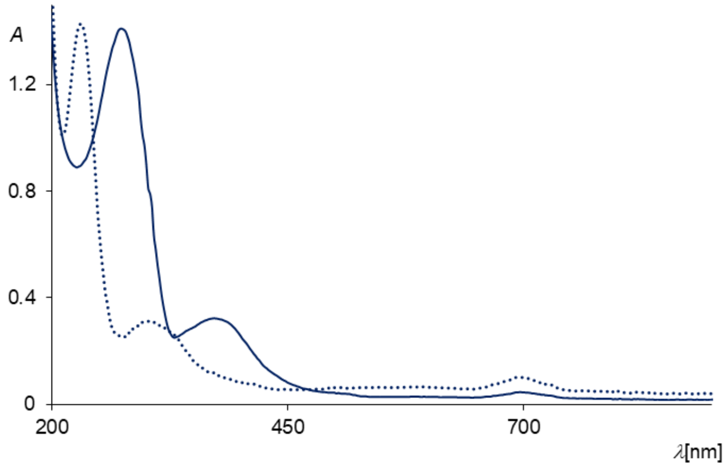

Figure S1. UV VIS NIR spectra of 10^{-3} mol·dm^{-3}: solid line - *trans*-[Mn^{III}(iso[14]aneN_4)Cl_2]Cl, dotted line - *cis*-[Mn^{III}([12]aneN_4)Cl_2]Cl in H_2O, 0.093 cm cell.

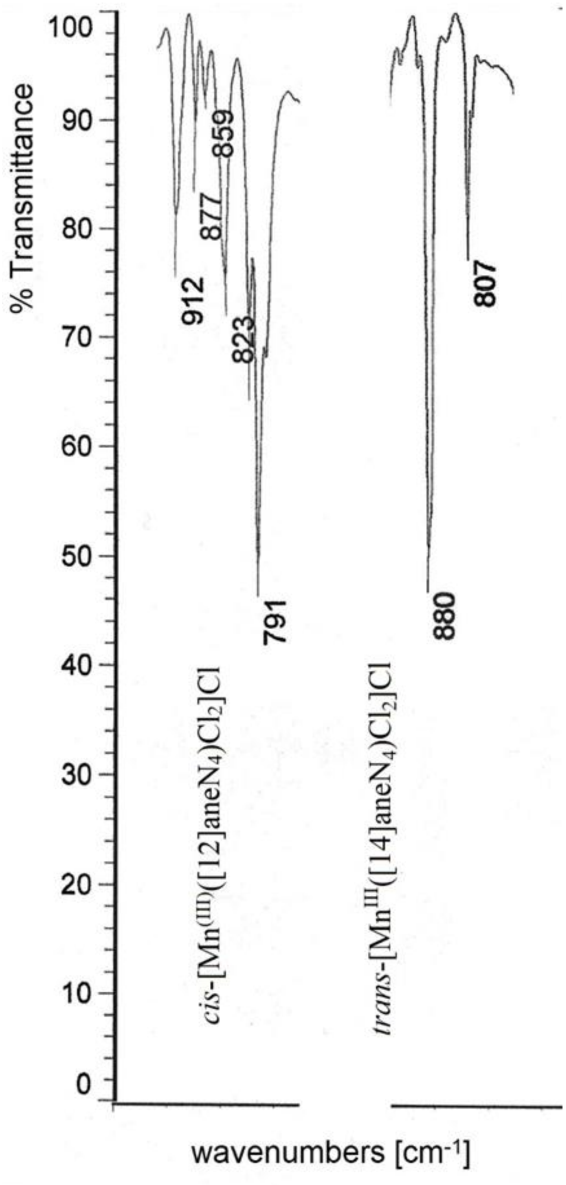

Figure S2. IR spectra of complexes: *trans*-[Mn^{III}([14]aneN_4)Cl_2]Cl and *cis*-[Mn^{III}([12]aneN_4)Cl_2]Cl in the range of 790-930 cm^{-1}, KBr pellet.

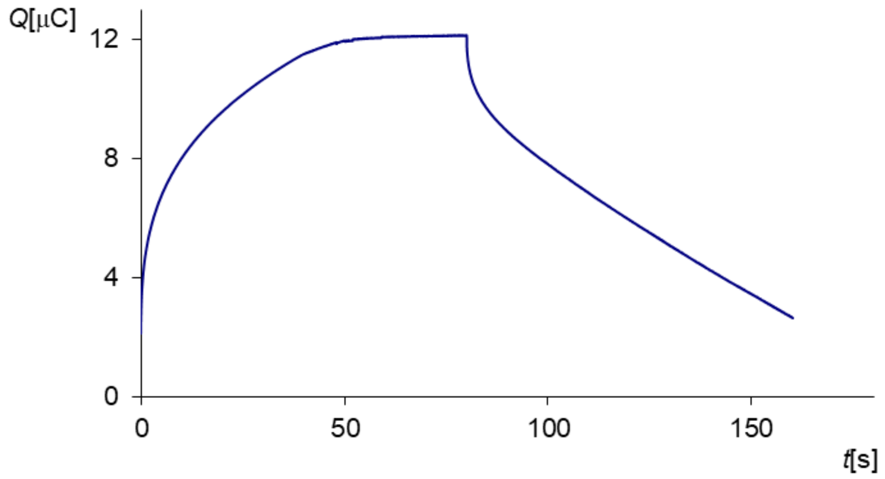

Figure S3. a) Chronocoulometric curve recorded for the first step of the electrode process on a glassy carbon disc electrode modified with the *trans*-[Mn^{III}(*iso* [14]aneN_4)Cl_2]Cl immobilized in Nafion (c = 0.062 mol·dm^3), μ = 0.1(KCl), pulse time = 80 s, step potential: -0.1 - 0.4 V, vs SCE.

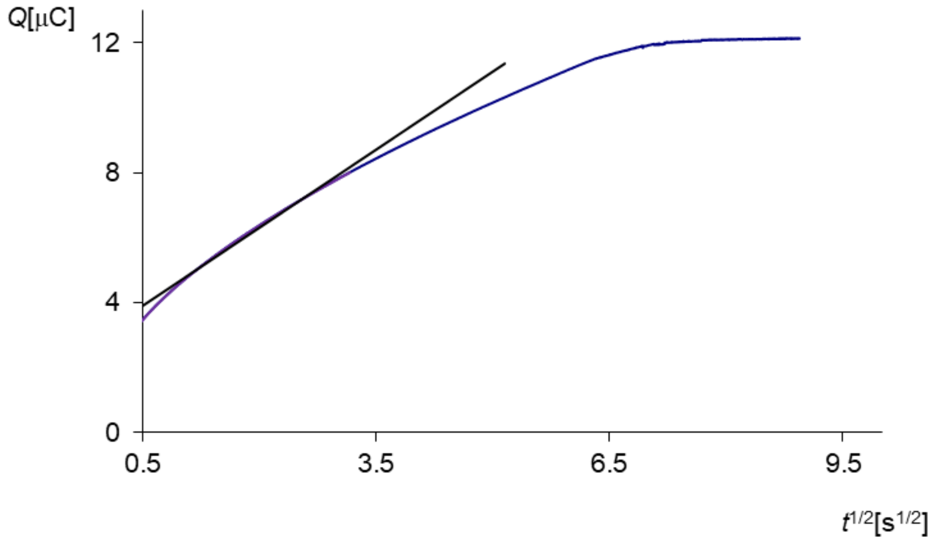

Figure S3. b) Q = f($t^{1/2}$) relationship curve for the oxidation process obtained from the chronocoulometric curve shown in Figure S3a.

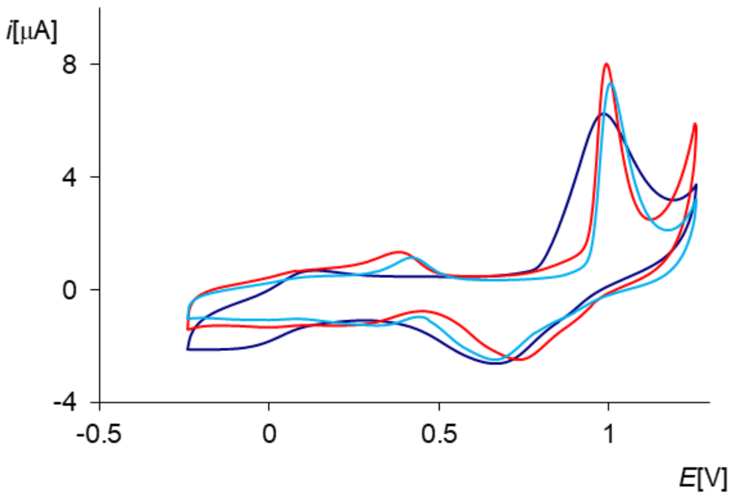

Figure S4. Cyclic voltammetry curves recorded on a glassy carbon disc electrode modified with the *trans*-$[Mn^{III}([15]aneN_4)Cl_2]$ complex immobilized in Nafion ($\Gamma = 6.7\cdot10^{-10}$ mol·cm^{-2}), navy line – in the absence of substrate, red line - in the presence of glycolic acid, blue line – in the presence of glycolic aldehyde (substrate concentration, $c = 10^{-3}$ mol·dm^{-3}), $\mu = 0.1$(KCl), v = 0.05 V·s^{-1}, vs SCE.

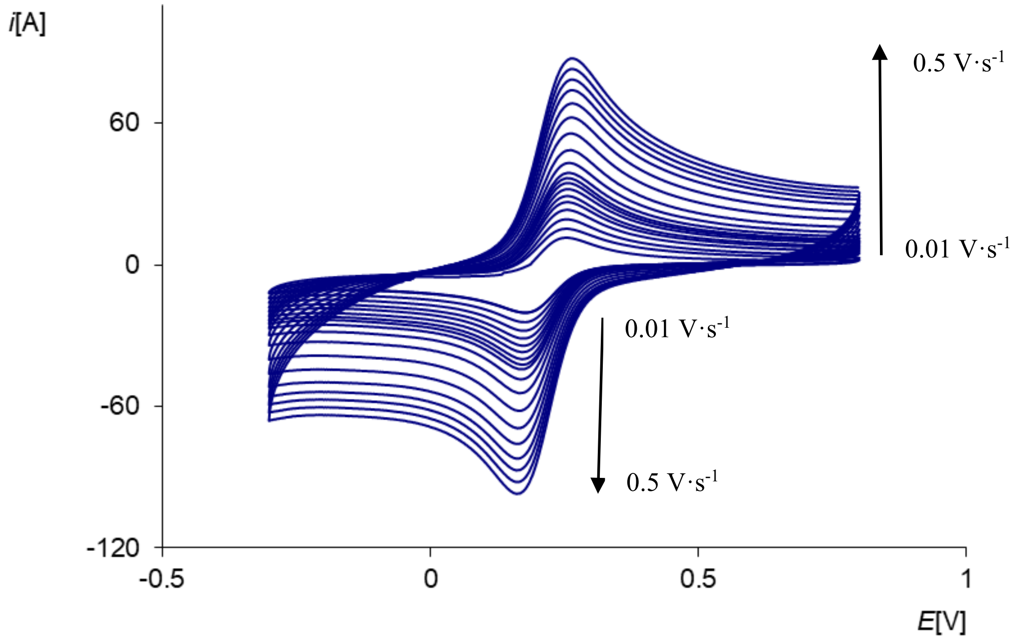

Figure S5. Cyclic voltammetry curves recorded on a glassy carbon disc electrode in the $K_3[Fe(CN)_6]$ solution, $c = 0.01$ mol·dm^{-3}, $\mu = 1$(KCl), at v: 0.01, 0.02, 0.03, 0.04, 0.05, 0.06, 0.07, 0.08, 0.09, 0.10, 0.12, 0.15, 0.20, 0.25, 0.30, 0.35, 0.40, 0.45 and 0.50 V·s^{-1}, vs SCE.

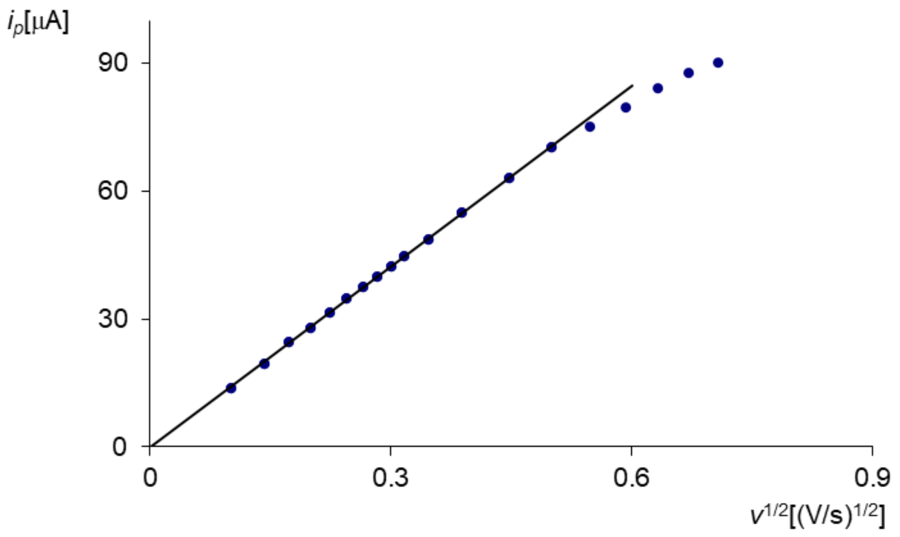

Figure S6. Dependence of cathode peak currents on the square root of the scan rate obtained from voltammograms recorded in the $K_3[Fe(CN)_6]$, c = 0.01 mol·dm^{-3}, μ = 1(KCl), glassy carbon disc electrode, vs SCE.

Tables

Table S1. Electrocatalytic activity of the studied complexes immobilized in Nafion (Γ = 1.5·10^{-9} mol·cm^{-2}) on the surface of a conventional glassy carbon electrode towards selected substrates (c = 10^{-3} mol·dm^{-3}), μ = 0.1(KCl), pulse time = 10 s, step potential: -0.1 - 1.2 V, vs SCE, determined on the basis of chronocoulometry.

Complex	Substrate	$Q_{a\,s}/Q_a$*
trans-[Mn^{III}([14]aneN$_4$)Cl$_2$]Cl	ascorbic acid<br>glycolic acid<br>glycolic aldehyde	1.67<br>1.59<br>1.46
trans-[Mn^{III}(*iso*[14]aneN$_4$)Cl$_2$]Cl	ascorbic acid<br>glycolic acid<br>glycolic aldehyde	1.70<br>1.59<br>1.49
cis-[Mn^{III}([12]aneN$_4$)Cl$_2$]Cl	ascorbic acid<br>glycolic acid<br>glycolic aldehyde	1.84<br>1.76<br>1.73
trans-[Mn^{III}([15]aneN$_4$)Cl$_2$]Cl	ascorbic acid<br>glycolic acid<br>glycolic aldehyde	1.45<br>1.37<br>1.32

* $Q_{a\,s})/Q_a$ - values of anodic charge, recorded in the presence of a substrate $(Q_{a\,s})$ to those recorded in the absence of a substrate (Q_p), charge read at 10 s.

Table S2. Electrocatalytic activity of the *trans*-[Mn^{III}(*iso*[14]aneN_4)Cl_2]Cl complex immobilized in a Nafion film on the surface of a conventional glassy carbon electrode against selected substrates ($c = 10^{-3}$ mol·dm^{-3}), depending on the complex concentration in the polymer film, $\mu = 0.1$(KCl), $v = 0.05$ V·s^{-1}, vs SCE., determined on the basis of voltammetry.

Complex	concentration of surface centers Γ [mol·cm^{-2}]	substrate	1st step processes		2nd step processes	
			$i_{pa\,S}/i_{pa}$*	$i_{pc\,R}/i_{pc}$*	$i_{pa\,S}/i_{pa}$*	$i_{pc\,S}/i_{pk}$*
trans-[Mn^{III}(*iso*[14]aneN_4)Cl_2]Cl	$6.7\cdot10^{-10}$	ascorbic acid	1.64	0.87	1.70	0.85
	$9.9\cdot10^{-10}$		1.63	0.87	1.68	0.85
	$1.5\cdot10^{-9}$		1.63	0.86	1.70	0.84
	$1.8\cdot10^{-9}$		1.64	0.85	1.68	0.83
	$2.2\cdot10^{-9}$		1.63	0.85	1.69	0.84
	$2.5\cdot10^{-9}$		1.62	0.86	1.68	0.84
	$6.7\cdot10^{-10}$	glycolic acid	1.56	0.87	1.62	0.86
	$9.9\cdot10^{-10}$		1.57	0.87	1.61	0.87
	$1.5\cdot10^{-9}$		1.57	0.86	1.62	0.86
	$1.8\cdot10^{-9}$		1.55	0.85	1.60	0.87
	$2.2\cdot10^{-9}$		1.55	0.86	1.60	0.85
	$2.5\cdot10^{-9}$		1.57	0.87	1.62	0.86
	$6.7\cdot10^{-10}$	glycolic aldehyde	1.48	0.91	1.53	0.89
	$9.9\cdot10^{-10}$		1.49	0.90	1.51	0.89
	$1.5\cdot10^{-9}$		1.50	0.91	1.52	0.90
	$1.8\cdot10^{-9}$		1.48	0.89	1.50	0.91
	$2.2\cdot10^{-9}$		1.48	0.90	1.52	0.89
	$2.5\cdot10^{-9}$		1.48	0.89	1.51	0.89

* $i_{pa\,S})/i_{pa}$ - values of anodic peak currents recorded in the presence of a substrate ($i_{pa\,S}$) to those recorded in the absence of a substrate (i_p); $i_{pc\,S})/i_{pc}$ - values of cathodic peak currents recorded in the presence of a substrate ($i_{pc\,S}$) to those recorded in the absence of a substrate (i_{pc}).

Table S3. Masses of conditioned Nafion films with complexes immobilized in their structures.

Complex	Concentration of the complex solution mixed with Nafion [mol·dm^{-3}]					
	$1\cdot10^{-2}$	$2\cdot10^{-2}$	$3\cdot10^{-2}$	$4\cdot10^{-2}$	$5\cdot10^{-2}$	$6\cdot10^{-2}$
	Mass of the conditioned Nafion film with the immobilized complex [g]					
trans-[Mn^{III}([14]aneN_4)Cl_2]Cl	0.0251	0.0253	0.0254	0.0256	0.0258	0.0259
trans-[Mn^{III}(*iso*[14]aneN_4)Cl_2]Cl	0.0251	0.0253	0.0255	0.0257	0.259	0.0260
cis-[Mn^{III}([12]aneN_4)Cl_2]Cl	0.0250	0.0252	0.0254	0.0256	0.258	0.0259
trans-[Mn^{III}([15]aneN_4)Cl_2]Cl	0.0252	0.0254	0.0256	0.0258	0.259	0.0260
